# Mechanically Robust and Conductive Gelatin/Glucose Hydrogels Enabled by the Hofmeister Effect for Flexible Strain Sensors

**DOI:** 10.3390/gels11090694

**Published:** 2025-09-01

**Authors:** Wei Sang, Xu Yang, Hui Li, Xiaoxu Liang, Hongyao Ding

**Affiliations:** 1 College of Intelligent Manufacturing, Yangzhou Polytechnic Institute, Yangzhou 225127, China; 2 College of Materials Science and Engineering, Nanjing Tech University, Nanjing 210009, China; 3 Key Laboratory for Light-Weight Materials, Nanjing Tech University, Nanjing 210009, China; 4 School of Arts and Sciences, Guangzhou Maritime University, Guangzhou 510725, China

**Keywords:** tough hydrogels, ionic conductive, Hofmeister effect

## Abstract

Conductive hydrogels are attractive for flexible electronics; however, achieving high mechanical strength and conductivity simultaneously remains challenging. Herein, we present a facile strategy to fabricate a tough and conductive hydrogel by immersing a physically crosslinked gelatin/glucose hydrogel in an aqueous sodium citrate. The introduction of sodium citrate induced multiple physical interactions via the Hofmeister effect, which synergistically reinforced the hydrogel network. The resulting hydrogel exhibited excellent mechanical properties, with a fracture strength of 2.7 MPa, a fracture strain of 932%, and a toughness of 9.5 MJ/m^3^. Moreover, the incorporation of free ions imparted excellent ionic conductivity of 0.97 S/m. A resistive strain sensor based on this hydrogel showed a linear and sensitive response over a wide strain range and stable performance under repeated loading–unloading cycles. These features enabled accurate and reliable monitoring of various human movements. This work offers an effective strategy for designing hydrogels with both high strength and conductivity for flexible and wearable electronics.

## 1. Introduction

With the rapid development of artificial intelligence, wearable electronic devices have attracted much attention in emerging fields such as flexible electronics, bioelectronics, and soft robotics [1,2,3]. Conductive hydrogels are considered promising candidates for these applications due to their excellent flexibility, biocompatibility, and stretchability [4,5]. Typically, conductive fillers incorporated into hydrogels include ionic and electronic conductive materials, such as inorganic salts, metallic nanomaterials, carbon-based materials, and conductive polymers [6,7,8,9,10]. However, traditional hydrogels still face critical challenges that limit their stability and service life, including high brittleness, poor toughness, low strength, and insufficient conductivity [11,12,13,14]. Therefore, it is essential to improve the mechanical strength and conductivity of hydrogels for advancing their practical applications in wearable electronics.

In recent years, various hydrogels with enhanced mechanical strength have been developed by introducing effective energy dissipation mechanisms into the gel network [15,16,17,18]. Among them, incorporating additional physical interactions, such as hydrogen bonding, electrostatic interaction, ionic interaction, hydrophobic association, and crystallization, has proven highly effective in reinforcing hydrogel mechanical properties [19,20,21,22,23]. For example, Yang et al. employed a strong acid-assisted training strategy to improve the mechanical properties of sodium polyacrylate hydrogel by constructing multiple dynamic hydrogen bonds [24]. Similarly, Li et al. fabricated the PVA-SA-PPy hydrogels via a repeated freeze–thaw process, which induced crystallization of PVA chains and promoted the hydrogen bonds formation between PVA and SA chains, thereby enhancing the mechanical properties of the hydrogels [25]. However, some of these methods were often complicated and time-consuming [26,27]. Therefore, developing a simple and feasible approach for synthesizing conductive hydrogels remains of great significance.

Immersion in salt solutions has emerged as a universal strategy for synthesizing tough and conductive hydrogels, primarily due to the salting out effect on polymers. This process enhances hydrogel toughness by promoting hydrophobic interactions or inducing the formation of microcrystal within the polymer network. Compared with methods that relied on multiple chemical reactions or complex fabrication steps, the soaking strategy was more convenient and efficient [28,29,30]. During the soaking process, the pre-formed hydrogel underwent dehydration caused by the salting out effect, a phenomenon widely used to explain the precipitation of proteins from solutions containing kosmotropic ions. Previous studies have demonstrated that hydrophobic interactions and chain bundles arising from chain aggregation played key roles in reinforcing the hydrogel network [31,32,33]. In addition, the incorporation of free ions from the salt solution significantly improved the electrical conductivity of the hydrogel. However, many existing methods rely on toxic reagents, complex synthesis, or high costs, which hinders the development of tough and conductive hydrogels from materials that are simple, readily available, low-cost, and biocompatible.

Gelatin, a natural biopolymer derived from collagen, was particularly attractive for hydrogel systems owing to its unique thermally reversible gelation behavior, which enabled the formation of physically crosslinked triple-helix structures at low temperatures [34,35]. Sodium citrate (SC) was a biocompatible, strong salting out agent in the Hofmeister series [36]. Herein, we report the fabrication of a mechanically robust and conductive hydrogel via a two-step strategy. Firstly, a gelatin/glucose hydrogel was prepared at low temperature. Subsequently, the hydrogel was immersed in a SC solution. During this process, the Hofmeister effect promoted the formation of additional triple-helices structures within the gelatin network, generating more hydrophobic domains and chain bundles. Simultaneously, it would strengthen the hydrogen bonding interactions between glucose and gelatin. These physical interactions can dissipate energy under loading, thus improving the mechanical properties of the hydrogels. As a result, the GU-S hydrogel exhibited excellent mechanical performance, including high strength and toughness. Benefiting from the abundant free ions within the network, the hydrogel also demonstrated superior ionic conductivity, making it particularly suitable for strain-sensing applications. The resulting hydrogel-based strain sensor showed high sensitivity, good linearity, a wide sensing window, and outstanding stability, enabling reliable monitoring of diverse human motions. This work presents a simple yet effective approach to constructing tough, conductive hydrogels with promising potential for wearable electronics.

## 2. Results and Discussion

### 2.1. Synthesis of Tough Hydrogels

**Figure 1:**
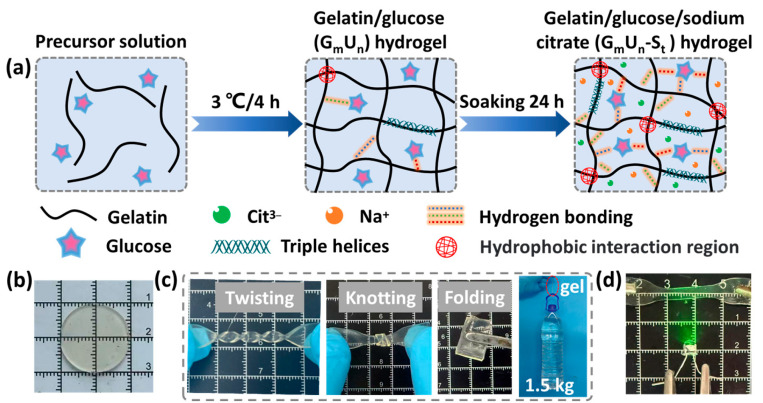
(**a**) Schematic illustration of the preparation process of G_m_U_n_-S_t_ hydrogel. (**b**) Picture of the G_220_U_300_-S_20_ hydrogel. (**c**) Demonstration of the mechanical flexibility of G_220_U_300_-S_20_ hydrogel under various deformations, including twisting, knotting, folding, and lifting a 1.5 kg bottle. (**d**) Photograph showing the G_220_U_300_-S_20_ hydrogel powering a LED.

A physically crosslinked tough and conductive hydrogel was prepared via a two-step method. The specific preparation process is shown in Figure 1a. Specifically, a mixture containing gelatin and glucose was injected into a mold and maintained at a low temperature for 3 h to obtain the as-prepared gelatin/glucose (G_m_U_n_) hydrogel. The G_m_U_n_ hydrogel was immersed in an aqueous sodium citrate (SC) solution at a specific concentration for 24 h to obtain the final G_m_U_n_-S_t_ hydrogel. The resulting G_m_U_n_-S_t_ hydrogels were all transparent with a slightly pale gray color (Figure 1b). The raw materials, including the gelatin, glucose, and SC, used in this preparation were all biocompatible, and the method was simple and easy to implement. The incorporation of glucose provided abundant hydrogen-bonding sites, thereby significantly enhancing the crosslinking density of the gel network. During the soaking process, the Hofmeister effect induced by SC facilitated the formation of more triple-helix structures among gelatin chains, as well as enhanced hydrogen bonding between gelatin and glucose. These multiple physical interactions can dissipate energy under loading, thus improving the mechanical properties of the hydrogel. As shown in Figure 1c, the obtained G_220_U_300_-S_20_ hydrogel withstood various deformations, including twisting, knotting, and folding. A small hydrogel sample with dimensions of 16 mm × 4 mm × 0.6 mm was able to support a load of 1.5 kg. In addition, the soaking process introduced a large number of free ions (Na^+^ and Cit^3−^) into the gel network, endowing the gel excellent ionic conductivity. This enabled the hydrogel to function as a conductor capable of powering a LED in a circuit (Figure 1d).

### 2.2. Structural Characterizations

**Figure 2:**
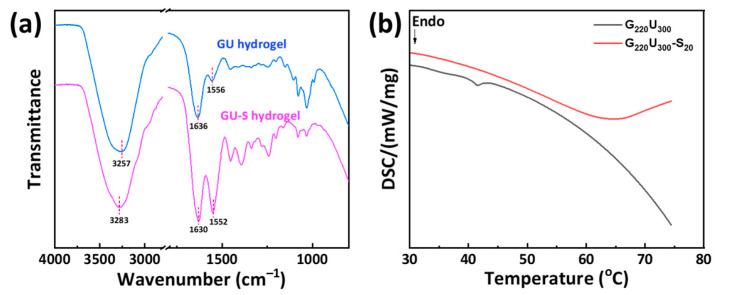
(**a**) FTIR spectra of G_220_U_300_ and G_220_U_300_-S_20_ hydrogels. (**b**) DSC curves of G_220_U_300_ and G_220_U_300_-S_20_ hydrogels.

Infrared spectroscopy was used to analyze the molecular structures of various hydrogels. In the spectrum of glucose (Figure S1), the strong absorption peak at 1032 cm^−1^ was attributed to the C–O stretching vibration in the glucose molecular backbone, while the broad absorption band at 3270 cm^−1^ corresponded to the O–H stretching vibration [37]. In the spectrum of gelatin, characteristic absorption peaks were observed at 1642 and 1555 cm^−1^, corresponding to the stretching vibrations of amide I (C=O) and amide II (N-H), respectively; an absorption band at 3331 cm^−1^ was assigned to the overlapping the stretching vibrations of N–H and O–H [38]. In the spectrum of G_220_U_300_ hydrogel (Figure 2a), the absorption peaks assigned to gelatin and glucose exhibited no significant shifts, indicating that the molecular structures of gelatin and glucose remained unchanged after physical mixing. Upon immersing the GU hydrogel in SC solution to obtain the GU-S hydrogel, the O–H/N–H stretching band, initially appearing as a broad feature centered at 3257 cm^−1^, exhibited a pronounced enhancement in intensity around 3283 cm^−1^. Meanwhile, the amide I and amide II bands shifted slightly from 1636 to 1630 cm^−1^ and from 1556 to 1552 cm^−1^, respectively. Moreover, the absorption band at 1030 cm^−1^ decreased in intensity. These spectral changes suggested that the SC facilitated the formation of more hydrogen bonds and triple-helix structures within the gel network, resulting in a more compact gel matrix [39].

DSC was employed to investigate the thermal behaviors of various hydrogels. As shown in Figure 2b, an obvious endothermic peak was observed at 41 °C on the DSC curve of G_220_U_300_ hydrogel, which can be mainly attributed to the disruption of hydrogen bonds and the variation of triple-helix structures. Notably, the temperature of this endothermic peak was significantly higher than that reported for pure gelatin hydrogel [40], which was ascribed to the formation of more stable hydrogen bonds between gelatin and glucose. After the introduction of SC, a distinct endothermic peak appeared at 64 °C on the DSC curve of G_220_U_300_-S_20_ hydrogel. This result indicated that the thermal stability of the hydrogel was effectively enhanced by the soaking strategy. The incorporation of SC promoted the formation of more stable hydrogen bonds and triple-helix structures within the gel network through the salting out effect, resulting in a more stable hydrogel network. The physical crosslinking arising from the salting out effect was relatively unstable and could be readily disrupted upon immersion in aqueous solutions. Consequently, as shown in Figure S2, the G_220_U_300_-S_20_ hydrogel exhibited rapid swelling in both deionized water and acidic aqueous solutions.

### 2.3. Mechanical Properties

**Figure 3:**
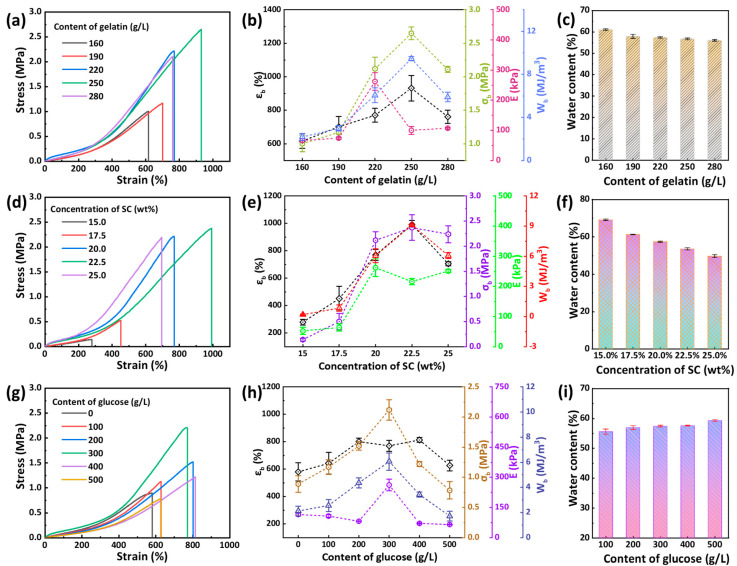
(**a**) Tensile stress–strain curves of G_m_U_300_-S_20_ hydrogels with different gelatin contents, (**b**) corresponding mechanical properties, and (**c**) their water content. (**d**) Tensile stress–strain curves of G_220_U_300_-S_t_ hydrogels with different SC concentrations, (**e**) corresponding mechanical properties, and (**f**) their water content. (**g**) Tensile stress–strain curves of G_220_U_n_-S_20_ hydrogels with different glucose contents, (**h**) corresponding mechanical properties, and (**i**) their water content.

The mechanical properties of the G_m_U_n_-S_t_ hydrogel were systematically investigated by uniaxial tensile tests. First, the effect of gelatin content (*C*_gelatin_) on the mechanical performance of G_m_U_300_-S_20_ hydrogel was analyzed. As shown in Figure 3a,b, with the increasing *C*_gelatin_ from 160 g/L to 250 g/L, the breaking stress (*σ_b_*), breaking strain (*ε_b_*), Young’s modulus (*E*), and toughness (*W*_b_) of the obtained gel increased from 1.0 MPa, 616%, 66 kPa, and 2.2 MJ/m^3^ to 2.7 MPa, 932%, 100 kPa, and 9.5 MJ/m^3^, respectively. A higher gelatin content promoted the formation of more physical interactions between polymer chains, which could increase the energy dissipation under loading. These physical interactions facilitated the formation of a more compact gel matrix, which can be reflected by their water content that showed a downward trend (Figure 3c). However, with a further increase in *C*_gelatin_ reaching 280 g/L, *σ_b_*, *ε_b_*, *E*, and *W*_b_ decreased to 2.1 MPa, 761%, 107 kPa, and 5.9 MJ/m^3^, respectively. This might be due to the insufficient solute content in the soaking solution. In addition, the mechanical properties of the G_220_U_300_-S_t_ hydrogels with different SC concentrations (*C*_SC_) were also investigated, as shown in Figure 3d,e. As the *C*_SC_ increased from 15 wt% to 22.5 wt%, *σ_b_*, *ε_b_*, *E*, and *W*_b_ increased significantly from 0.1 MPa, 279%, 52 kPa, and 0.2 MJ/m^3^ to 2.4 MPa, 992%, 216 kPa, and 9.1 MJ/m^3^, respectively. On the contrary, the water content of these gels showed a downward trend (Figure 3f). The mechanical improvement was attributed to the enhanced Hofmeister effect at higher SC concentrations, which promoted the formation of more physical interactions inside the gel matrix. However, as *C*_SC_ increased to 25 wt%, *σ_b_*, *ε_b_*, and *W*_b_ of the gel decreased to 2.2 MPa, 705%, and 6.1 MJ/m^3^, respectively. This might be attributed to the enhanced alkalinity of the soaking solution at excessively high SC content, which compromised the stability of hydrogen bonds in the gel network. Moreover, the effect of the glucose content (*C*_glucose_) on the mechanical properties of the G_220_U_n_-S_20_ hydrogels was investigated, as shown in Figure 3g,h. As *C*_glucose_ increased from 0 to 300 g/L, *σ_b_*, *ε_b_*, and *W*_b_ increased from 0.9 MPa, 580%, and 2.1 MJ/m^3^ to 2.1 MPa, 770%, and 6.1 MJ/m^3^, respectively. This enhancement was ascribed to the formation of additional hydrogen bonds between glucose molecules and gelatin network, thereby reinforcing the hydrogel structure. However, when *C*_glucose_ was further increased from 300 g/L to 500 g/L, *σ_b_*, *ε_b_*, and *W*_b_ decreased to 0.8 MPa, 627%, and 1.71 MJ/m^3^, respectively. With increasing the glucose content, the *E* fluctuated below 200 kPa, and the water content of these gels showed a trend of slow increase (Figure 3i). Based on the above results, the G_m_U_n_-S_t_ hydrogel demonstrated a combination of high strength, high toughness, high extensibility, and low modulus. Compared to other gelatin-based hydrogels (Table S1), the GU-S hydrogel prepared in this work exhibited certain advantages in mechanical properties.

**Figure 4:**
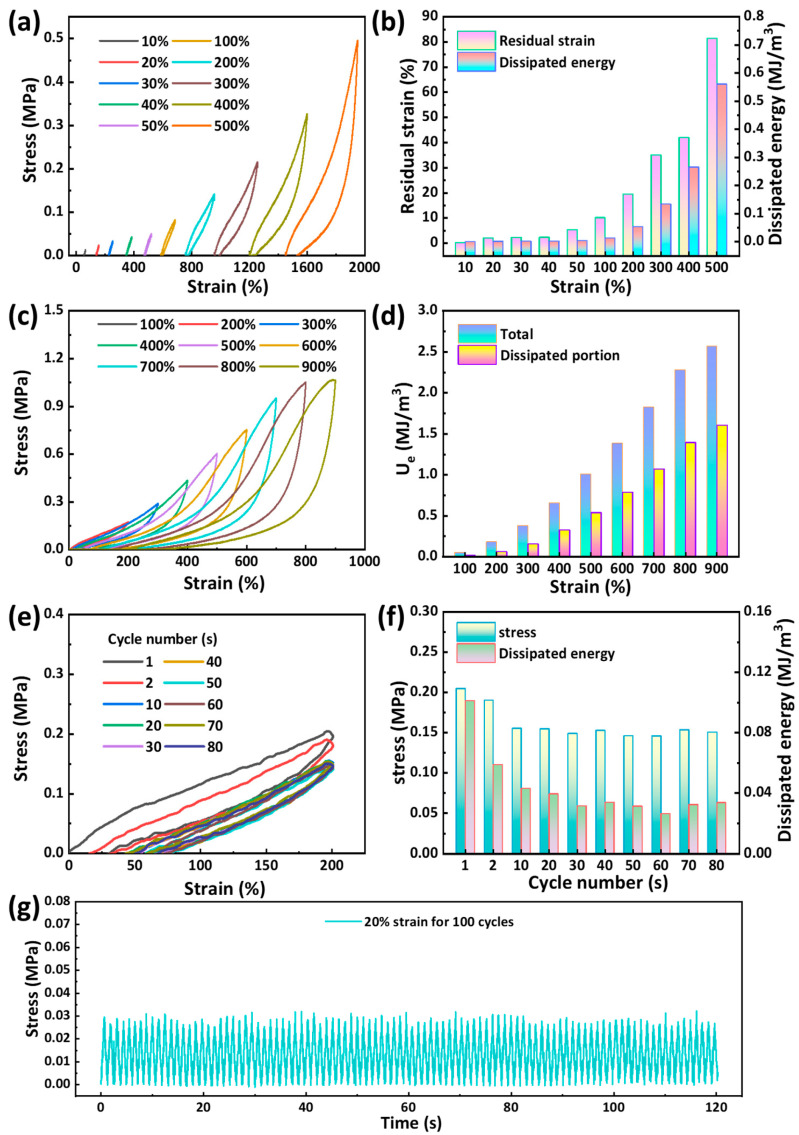
(**a**) Cyclic loading–unloading curves of G_220_U_300_-S_22.5_ hydrogels at different strains and (**b**) their corresponding residual strain and dissipated energy. (**c**) Cyclic loading–unloading curve of a single G_220_U_300_-S_22.5_ hydrogel with the strain ranging from 100% to 900% and (**d**) their corresponding total toughness and dissipated energy. (**e**) Loading–unloading curves over 80 cycles at a fixed strain of 200% and (**f**) their corresponding maximum stress and dissipated energy. (**g**) Stress–time curves of the G_220_U_300_-S_22.5_ hydrogel over 100 loading–unloading cycles at a maximum strain of 20%.

In addition, we also investigated the mechanical properties of the gel through cyclic experiments. A series of cyclic loading–unloading tests was performed on hydrogel of G_220_U_300_-S_22.5_, with the maximum strain ranging from 10% to 500% (Figure 4a,b). As the maximum strain increased from 10% to 50%, the residual strain and dissipated energy increased from 0.13% and 0.17 kJ/m^3^ to 5.3% and 3.7 kJ/m^3^, respectively. The loading and unloading curves nearly overlapped, indicating lower energy dissipation. However, as the maximum strain further increased from 100% to 500%, the residual strain and dissipated energy significantly increased from 10.1% and 12 kJ/m^3^ to 81.3% and 560 kJ/m^3^, respectively. The hysteresis loop in the stretching cycle process also gradually increased. This pronounced hysteresis was attributed to the intrinsic viscoelasticity of the polymer network [41]. To further investigate the energy dissipation behavior, a continuous cyclic loading–unloading test was conducted on a single G_220_U_300_-S_22.5_ hydrogel, with the maximum strain ranging from 100% to 900% (Figure 4c,d). The total toughness (U_e_) and dissipated energy at different strain were analyzed. With increasing applied strain, the hysteresis loops became progressively larger, demonstrating an increase in dissipated energy. This behavior indicated the disruption of multiple dynamic physical interactions within the hydrogel network. The significant overlap between adjacent loading–unloading curves suggested that some disrupted physical interactions can be rapidly reconstructed during unloading, demonstrating the hydrogel’s rapid self-recovery capability. In addition, 80 consecutive loading–unloading cycles were performed on G_220_U_300_-S_22.5_ hydrogel at a fixed maximum strain of 200% (Figure 4e,f). The variations in maximum stress and energy dissipation gradually decreased after around the 10th cycle, indicating that the hydrogel structure had stabilized under repeated large deformations. Furthermore, after 100 loading–unloading cycles with a maximum strain of 20%, the cyclic stress–strain curves remained nearly unchanged (Figure 4g), demonstrating the hydrogel’s excellent fatigue resistance and structural stability at low strain.

### 2.4. Conductivity of G_m_U_n_-S_t_ Hydrogels

During the soaking process, a large number of free ions were introduced into the gel network, which migrated directionally under an applied electric field, thereby imparting good ionic conductivity to the hydrogel. The effects of hydrogel components and SC concentration on the ionic conductivity were investigated. As the *C*_gelatin_ increased from 160 g/L to 280 g/L, the corresponding conductivity of the GU-S hydrogels decreased from 1.86 S/m to 0.82 S/m (Figure S3a). Similarly, as the *C*_SC_ increased from 15 wt% to 25 wt%, the conductivity of the GU-S hydrogel decreased from 2.01 S/m to 0.41 S/m (Figure S3b). With the increase in *C*_gelatin_ and *C*_SC_, the hydrogel matrix became more compact, leading to a reduction in the effective pore size within the network, thereby decreasing the efficiency of ion migration [42]. In addition, as the *C*_glucose_ increased from 0 g/L to 300 g/L, the conductivity enhanced from 0.94 S/m to 1.43 S/m (Figure S3c), due to the increased water content that facilitated ion transport. However, a further increase in *C*_glucose_ from 300 g/L to 500 g/L resulted in a decline in ionic conductivity to 0.77 S/m. This could be attributed to the excessive presence of glucose molecules, which blocked the pore structure of hydrogel and therefore impeded the migration of free ions. The excellent ionic conductivity of these hydrogels enables their potential applications in the field of flexible sensing.

### 2.5. Sensing Performance and Application for Monitoring Human Motions

**Figure 5:**
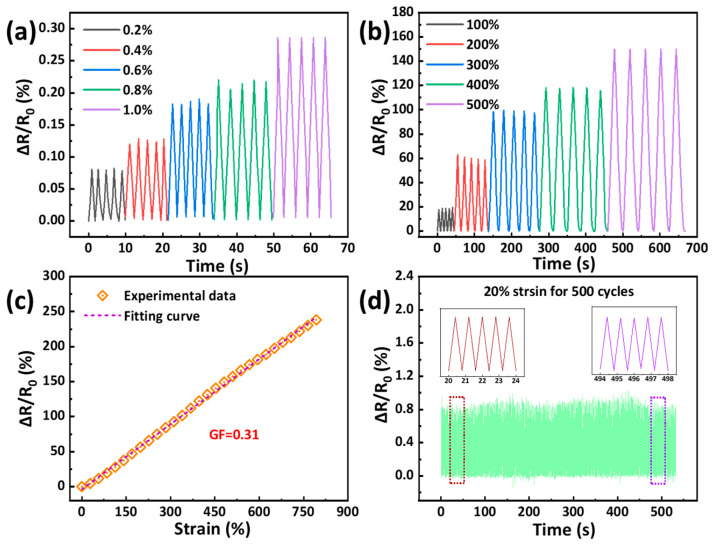
Relative resistance changes of the G_220_U_300_-S_22.5_ hydrogel sensors in response to (**a**) small strains (0.2–1%) and (**b**) large strains (100–500%). (**c**) Relationship between relative resistance change and strain of the hydrogel sensor. (**d**) Relative resistance change of the hydrogel sensor during 500 cyclic tests at a maximum strain of 20%.

As stated above, the GU-S hydrogels possessed high strength, high elongation, low modulus, and satisfactory ionic conductivity, facilitating their usage as flexible strain sensors. In this study, a resistive-type strain sensor was fabricated using the G_220_U_300_-S_22.5_ hydrogel, and its sensing performance was evaluated. As shown in Figure 5a,b, the hydrogel-based sensor was capable of detecting deformations under both small strains (0.2–1.0%) and large strains (100–500%), and these signal peaks were basically repeated under multiple strains, demonstrating its high sensitivity and wide sensing range. During stretching, the cross-sectional area of hydrogel decreased, which impeded the migration of free ions and led to an increase in resistance with the increasing strain. A plot of the relative resistance change (ΔR/R_0_) as a function of strain was obtained, and the corresponding gauge factor (GF) was calculated as 0.31 from the slope of the fitted curve (Figure 5c), indicating high sensitivity of the hydrogel sensor. In addition, the GF exhibited a linear dependence over a wide strain range, which can simplify data processing by eliminating the need for complex nonlinear fitting [43]. Compared to other hydrogel-based sensors (Table S1), the GU-S hydrogel sensors developed in this work exhibited certain advantages in comprehensive sensing performance. Furthermore, the hydrogel sensor displayed stable and repeatable electrical signals over 500 cycles under a maximum strain of 20% (Figure 5d), demonstrating its excellent durability and structural stability.

**Figure 6:**
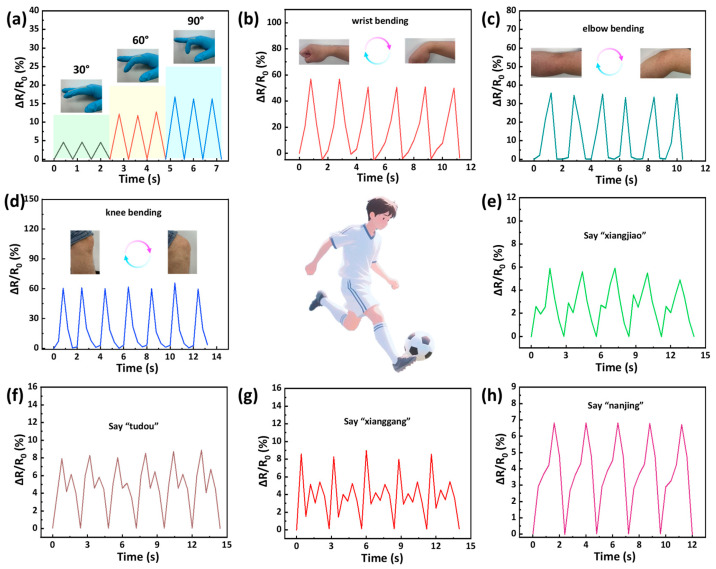
Real-time relative resistance changes of hydrogel sensors for monitoring both large and subtle human movements: (**a**) finger bending at various angles (30°, 60°, and 90°); (**b**) wrist bending; (**c**) elbow bending; and (**d**) knee bending. (**e**–**h**) The application of hydrogel-based sensors in detecting the pronunciation of various words.

The hydrogel-based sensor, featuring high sensitivity, a linear response over a wide strain range, and good stability, was well suited for applications in human motion monitoring. Its performance was evaluated by attaching the sensor to different parts of the human body. The sensor effectively detected finger bending, where distinct signal peaks corresponded to different bending angles (Figure 6a). The sensors also exhibited a reliable response to movements of other joints, including wrist, elbow, and knee bending (Figure 6b–d). Furthermore, the sensors were capable of capturing subtle throat vibrations during speech. Specifically, the pronunciations of different words generated distinct electrical signals, and these signals exhibited good repeatability (Figure 6f–h). These results indicated that the hydrogel sensors possessed high sensitivity and the capability to discriminate between different speech patterns. Therefore, the GU-S hydrogel-based sensors hold great potential for applications in motion detection devices and voice-interactive systems.

## 3. Conclusions

In summary, a mechanically robust and conductive GU-S hydrogel was successfully developed via a two-step method, in which a gelatin/glucose precursor solution was first gelled and subsequently soaked in a SC solution. This approach exploited the Hofmeister effect to induce additional triple-helix structure formation and strengthen the hydrogen bonding between gelatin and glucose. These non-covalent interactions served as energy dissipation mechanisms during deformation, significantly improving the mechanical performance of the hydrogel. The mechanical properties can be tuned by varying the hydrogel composition, yielding *σ_b_* ranging from 0.2 to 2.7 MPa, *ε_b_* from 279% to 992%, *E* from 52 to 262 kPa, and W_b_ from 0.2 to 9.2 MJ/m^3^. Moreover, the incorporation of free ions imparted excellent ionic conductivity to the hydrogel. When applied as a resistive strain sensor, the GU-S hydrogel demonstrated a broad sensing range (0–800% strain), excellent linearity, high sensitivity, and good cyclic stability over 500 loading–unloading cycles. The hydrogel sensors were capable of monitoring various human motions from large joint movements to subtle throat vibrations. Compared with reported gelatin-based conductive hydrogels, the GU-S hydrogels offer clear advantages in facile preparation, biocompatibility, and balanced mechanical-sensing performance. Nevertheless, their stabilities will decrease under harsh conditions such as extreme temperatures or prolonged aqueous exposure, leading to the loss of strength and conductivity. Incorporating anti-freezing and anti-dehydration strategies could enhance durability and expand their applications in wearable electronics.

## 4. Materials and Methods

### 4.1. Materials

Gelatin, glucose, and sodium citrate (≥98%, SC) were supplied by Shanghai Aladdin Biochemical Technology Co., Ltd. (Shanghai, China). All chemical reagents were used directly for the preparation of hydrogels without further purification. Millipore deionized water was used in all experiments.

### 4.2. Preparation of Hydrogels

Gelatin and glucose were added to a bottle filled with a certain amount of water, and the mixture was then heated at 60 °C until the gelatin was completely dissolved, forming a homogeneous solution. The resulting solution was ultrasonicated to remove bubbles and then injected into a homemade glass mold using a syringe. The mold was then placed in a refrigerator at 3 °C for 4 h to obtain the as-prepared gelatin/glucose hydrogel. Subsequently, the gelatin/glucose hydrogel was immersed in a SC solution with a specified concentration for 24 h to obtain the final gelatin/glucose/SC hydrogel. The resulting hydrogels were denoted as G_m_U_n_-S_t_, with m, n, and t indicating the concentrations of gelatin, glucose, and SC in the soaking solution, respectively. The concentrations of gelatin (160, 190, 220, 250, and 280 g/L); glucose (100, 200, 300, 400, and 500 g/L); and SC (15.0, 17.5, 20.0, 22.5, and 25.0 wt%) were systematically varied.

### 4.3. Structural and Performance Characterizations

Tensile tests were conducted at room temperature (25 °C) using an electronic universal testing machine (UTM2502, SUNS Technology Stock Co., Ltd., Shenzhen, China) to evaluate the mechanical properties of hydrogels. The specimen was cut into a dumbbell shape with an initial gauge length of 16 mm and width of 4 mm. The thickness of the tested sample was measured using a thickness gauge prior to testing. During testing, the lower fixture was fixed while the upper fixture was displaced upward at a constant rate of 100 mm/min until the specimen fractured. Tensile stress (*σ*) was defined as the applied force divided by the initial cross-sectional area. Tensile strain (*ε*) was defined as the displacement of the upper fixture divided by the initial gauge length of the specimen. Breaking stress and breaking strain were denoted as *σ_b_* and *ε_b_*, respectively. Tensile Young’s modulus (*E*) was determined from the slope of the linear region of the tensile stress–strain curve. Toughness (*W*_b_) was calculated as the area under the stress–strain curve. In the cyclic loading–unloading tests, specimens were stretched to a specified strain at a tensile speed of 100 mm/min and then unloaded to the initial strain with the same rate. Dissipated energy was calculated from the area enclosed between the loading and unloading curves.

The molecular structures of various hydrogels were characterized using a Fourier-transform infrared spectrometer (ATR-FTIR, Bruker Vector 22, Bruker Corporation, Bremen, Germany) with a resolution of 4 cm^−1^ and a spectral range of 4000–600 cm^−1^.

A differential scanning calorimeter (DSC 300, NETZSCH, Waldkraiburg, Germany) was used to investigate the thermostability of GU and GU-S hydrogels. The measurements were conducted in the temperature range of 30–75 °C at a heating rate of 2 °C/min under a nitrogen atmosphere.

The conductivity of the hydrogel was measured using an electrochemical workstation (CH Instruments, CHI650E, Austin, TX, USA) via the AC impedance method. Two platinum sheet electrodes (1 cm × 1 cm × 0.2 mm) were connected to the workstation via copper wires. The hydrogel sample was assembled into a sandwich configuration between the platinum electrodes. Conductivity was calculated according to the reported references [44,45].

Two ends of long strip hydrogel were connected to a multimeter (Keysight, 34465A, Keysight Technologies, Inc., Santa Rosa, CA, USA) for sensing performance evaluation. A mechanical testing system was employed to control the cyclic stretching-releasing process. Real-time resistance during deformation was recorded, with relative resistance change (ΔR/R_0_) calculated according to the reported references [46,47].

## Data Availability

The original contributions presented in this study are included in the article/Supplementary Materials. Further inquiries can be directed to the corresponding author(s).

## Supplementary Materials

The following supporting information can be downloaded at https://www.mdpi.com/article/10.3390/gels11090694/s1. Figure S1. FTIR spectra of glucose and gelatin hydrogels.; Figure S2. Swelling ratio of G220U300-S20 hydrogels in both deionized water and acidic aqueous solutions; Figure S3. Electrical conductivity of (a) GmU300-S20 hydrogels with different gelatin content, (b) G220U300-St hydrogels with different SC concentration, and (c) G220Un-S20 hydrogels with different glucose contents; Table S1 Comparison of performance with other hydrogels [48,49,50,51,52,53,54,55,56,57].
