# Supplementary material for: Mechanically Robust and Conductive Gelatin/Glucose Hydrogels Enabled by the Hofmeister Effect for Flexible Strain Sensors
Source: Gels. 2025 Sep 1;11(9):694. doi: 10.3390/gels11090694 (PMC12469848; doi:10.3390/gels11090694)
Supplement: Supplementary file 1 [file gels-11-00694-s001.zip › gels-3823848-supplementary.pdf]

Supporting Information

Mechanically Robust and Conductive Gelatin/Glucose Hydrogels Enabled by Hofmeister Effect for Flexible Strain Sensor

Wei Sang,[1] Xu Yang,[2,3] Hui Li,[3] Xiaoxu Liang,[4,*] Hongyao Ding [2,*]

[1] School of Intelligent Manufacturing, Yangzhou Polytechnic Institute, Yangzhou 225127, China.
[2] College of Materials Science and Engineering, Nanjing Tech University, Nanjing, 210009, China.
[3] Key Laboratory for Light-weight Materials, Nanjing Tech University, Nanjing 210009, China.
[4] School of Arts and Sciences, Guangzhou Maritime University, Guangzhou 510725, China.
[*] Correspondence: E-mail: liangxxu@126.com (X. Liang), hongyaoding@njtech.edu.cn (H. Ding)

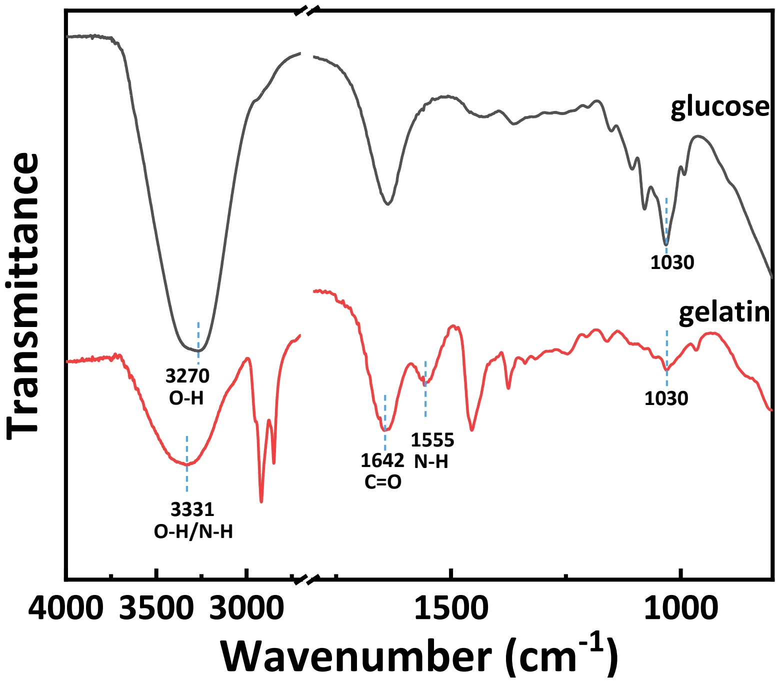

Figure S1. FTIR spectra of glucose and gelatin hydrogels.

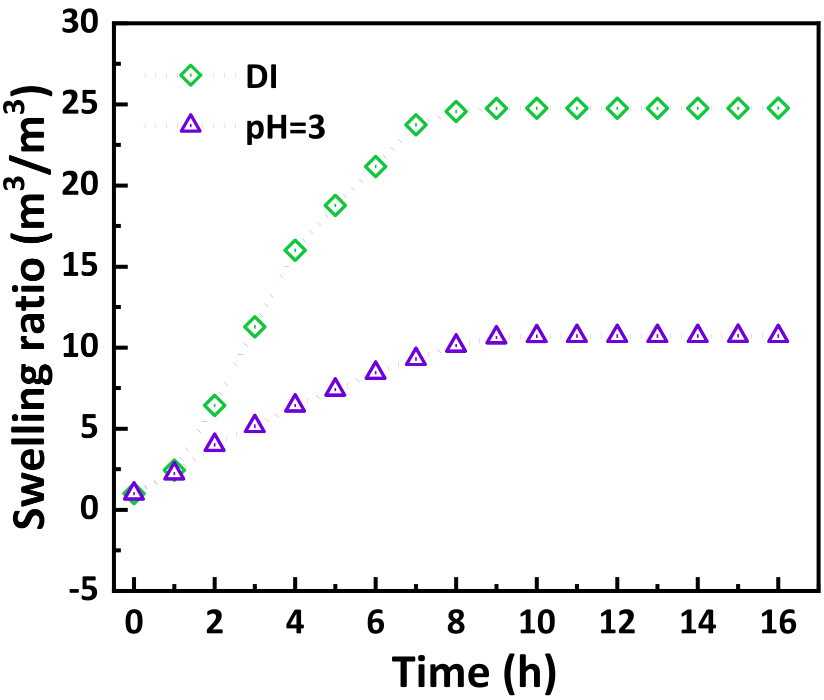

Figure S2. Swelling ratio of $G_{220}U_{300}$-S_{20} hydrogels in both deionized water and acidic aqueous solutions.

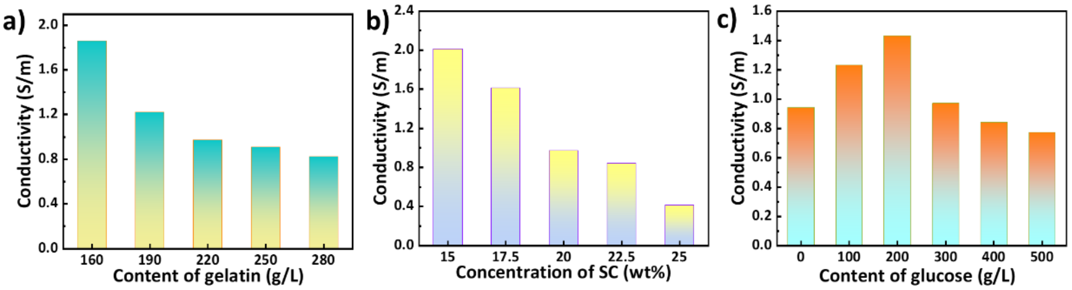

Figure S3. Electrical conductivity of (a) G_mU_{300}-S_{20} hydrogels with different gelatin content, (b) $G_{220}U_{300}$-S_t hydrogels with different SC concentration, and (c) $G_{220}U_n$-S_{20} hydrogels with different glucose contents.

Table S1 Comparison of performance with other hydrogels.

Hydrogel	Stress (MPa)	Strain (%)	Sensing window	Linearity	References
gelatin/O-dextran	2.49	570	0-200%	linear	[1]
gelatin/PPy/PAM/TA	1.44	470	0-450%	Piecewise linear	[2]
gelatin/GO/SA	1.7	90	0-35%	Piecewise linear	[3]
gelatin/PVA-$FeCl_3$	2.5	260	0-300%	linear	[4]
gelatin/OCTN/CMX	1.55	324	0-180%	Piecewise linear	[5]
gelatin/PAM/PA	0.1	1320	0-900%	Nonlinear	[6]
gelatin/CNF/PVCIF	1.28	327	0-350%	Piecewise linear	[7]
gelatin/TA/S-80	0.17	404	0-260%	Piecewise linear	[8]
gelatin/PAM/LiCl/gly	0.12	400	0-200%	Piecewise linear	[9]
gelatin/PVA/TT/CNT	0.12	389	0-760%	linear	[10]
gelatin/glucose-SC	2.65	932	0-800%	linear	This work

References

[1] L. Cao, Z. Zhao, X. Wang, X. Huang, J. Li, Y. Wei, Tough, antifreezing, and conductive hydrogel based on gelatin and oxidized dextran, Advanced Materials Technologies 7(7) (2022) 2101382.

[2] H. Qie, Z. Wang, J. Ren, S. Lü, M. Liu, A tough shape memory hydrogel strain sensor based on gelatin grafted polypyrrole, Polymer 263 (2022) 125524.

[3] R. Yin, C. Zhang, Y. Chen, Y. Wang, Q. Feng, Y. Liu, M. Yu, Y. Yuan, C.-Y. Xu, F. Liu, Transient, printable and recyclable gelatin hydrogels with enhanced mechanical sensing and electromagnetic shielding performance by incorporation of reduced graphene oxide, Chemical

Engineering Journal 475 (2023) 145794.
[4] S. Sun, Y. Xu, X. Maimaitiyiming, Tough polyvinyl alcohol-gelatin biological macromolecules ionic hydrogel temperature, humidity, stress and strain, sensors, International Journal of Biological Macromolecules 249 (2023) 125978.
[5] J. Zhu, H. Xu, Q. Hu, Y. Yang, S. Ni, F. Peng, X. Jin, High stretchable and tough xylan-g-gelatin hydrogel via the synergy of chemical cross-linking and salting out for strain sensors, International Journal of Biological Macromolecules 261 (2024) 129759.
[6] W. Li, Y. Ming, L. Yang, Y. Ni, Y. Chen, W. Xu, L. Li, C. Zheng, W. Lin, Conductive Hydrogel Motion Sensor with Low-Temperature Stability for Winter Sports and Sensing Rescue, Polymers 17(10) (2025) 1365.
[7] W. Song, H. Chen, P. Lu, Z. Miao, Y. Zhao, Z. He, Z. Ren, V. Nica, L. Qian, Polymeric ionic liquid modifier as ion-induced crosslinker and functional enhancer: Facile fabrication of multifunctional gelatin hydrogels for flexible electronics, International Journal of Biological Macromolecules 318 (2025) 145296.
[8] X. Ma, W. Xu, J. Chen, Y. Wang, W. Xiong, J. Li, L. You, S. Wang, Breathable Gelatin Conductive Hydrogels Using a Template Method and Reverse Use of Hofmeister Effect for Wearable Sensors, ACS Applied Polymer Materials 6(11) (2024) 6290-6301.
[9] L. Xu, X. Li, J. Gao, M. Yan, Q. Wang, Environment-tolerant gelatin based ionic conductive organohydrogel for flexible sensor, Materials Today Communications 40 (2024) 109542.
[10] Z. He, J. Liu, X. Fan, B. Song, H. Gu, Tara tannin-cross-linked, underwater-adhesive, super self-healing, and recyclable gelatin-based conductive hydrogel as a strain sensor, Industrial & Engineering Chemistry Research 61(49) (2022) 17915-17929.
